# Physiology of the widespread pulsating soft coral *Xenia umbellata* is affected by food sources, but not by water flow

**DOI:** 10.1002/ece3.10483

**Published:** 2023-09-01

**Authors:** C. E. L. Hill, S. G. Abbass, G. Caporale, Y. C. El‐Khaled, L. Kuhn, T. Schlenzig, C. Wild, A. Tilstra

**Affiliations:** ^1^ Marine Ecology Department, Faculty of Biology and Chemistry University of Bremen Bremen Germany; ^2^ Marine Science Department, Faculty of Science Port Said University Port Said Egypt; ^3^ Red Sea Research Center, Biological and Environmental Science and Engineering Division King Abdullah University of Science and Technology Jeddah Saudi Arabia

**Keywords:** carbon, current regime, nitrogen, phytoplankton, trophic ecology

## Abstract

Coral energy and nutrient acquisition strategies are complex and sensitive to environmental conditions such as water flow. While high water flow can enhance feeding in hard corals, knowledge about the effects of water flow on the feeding of soft corals, particularly those pulsating, is still limited. In this study, we thus investigated the effects of feeding and water flow on the physiology of the pulsating soft coral *Xenia umbellata*. We crossed three feeding treatments: (i) no feeding, (ii) particulate organic matter (POM) in the form of phytoplankton and (iii) dissolved organic carbon (DOC) in the form of glucose, with four water volume exchange rates (200, 350, 500 and 650 L h^−1^) over 15 days. Various ecophysiological parameters were assessed including pulsation rate, growth rate, isotopic and elemental ratios of carbon (C) and nitrogen (N) as well as photo‐physiological parameters of the Symbiodiniaceae (cell density, chlorophyll‐*a* and mitotic index). Water flow had no significant effect but feeding had a substantial impact on the physiology of the *X. umbellata* holobiont. In the absence of food, corals exhibited significantly lower pulsation rates, lower Symbiodiniaceae cell density and lower mitotic indices compared to the fed treatments, yet significantly higher chlorophyll‐*a* per cell and total N content. Differences were also observed between the two feeding treatments, with significantly higher pulsation rates and lower chlorophyll‐*a* per cell in the DOC treatment, but higher C and N content in the POM treatment. Our findings suggest that the *X. umbellata* holobiont can be viable under different trophic strategies, though favouring mixotrophy. Additionally, the physiology of the *X. umbellata* may be regulated through its own pulsating behaviour without any positive or negative effects from different water flow. Therefore, this study contributes to our understanding of soft coral ecology, particularly regarding the competitive success and widespread distribution of *X. umbellata*.

## Graphical abstract

This manuscript reports on the separate and interactive effects of varying water flow regimes and feeding on the physiology of the soft coral *Xenia umbellata*. From our study, we concluded that *X. umbellata* does not receive any additional benefit from high water flow nor suffer in low flow conditions, as it may regulate autotrophy and heterotrophy via the continuous pulsation of its polyps. Furthermore, findings suggest that photosynthetic energy generation of the *X. umbellata* holobiont is enhanced via increased chlorophyll‐*a* contents per cell when food is scarce. The findings of our study contribute toward understanding the ecology of soft corals and offer an additional explanation for the widespread distribution and competitive success of the mixotrophic *X. umbellata*.

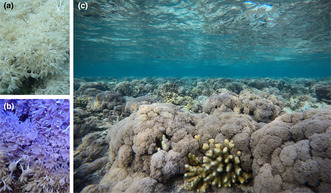

## 1 INTRODUCTION

Tropical coral reefs are highly productive and host a huge diversity of organisms. However, they inhabit oligotrophic waters, which are deficient in essential nutrients. Therefore, corals rely on several key mechanisms of nutrient and energy acquisition such as autotrophy (Muscatine & Porter, 1977) and heterotrophy (Houlbrèque & Ferrier‐Pagès, 2009) in order to meet their metabolic demands. Corals exist on a spectrum where their individual dependence on autotrophy and heterotrophy differs between species. Some corals are purely autotrophic or heterotrophic while others are mixotrophic and derive nutrition from both feeding modes (Conti‐Jerpe et al., 2020; Fabricius & Klumpp, 1995; Fox et al., 2018; Sturaro et al., 2021).

Autotrophy in corals is facilitated by their symbiotic relationship with Symbiodiniaceae that photosynthesise and translocate carbon to the coral host (LaJeunesse et al., 2018; Muller‐Parker et al., 2015). The rate of carbon fixation by Symbiodiniaceae is high, and fundamental amino acids and sugars can be assimilated by the coral within a matter of seconds (Streamer et al., 1993). Research has even shown that healthy corals that harbour Symbiodiniaceae are able to meet 100% of their daily metabolic demand via autotrophy alone (Grottoli et al., 2006). Corals can also feed heterotrophically, by actively preying on dissolved (DOM) and particulate organic matter (POM) of varying size classes (Houlbrèque & Ferrier‐Pagès, 2009), to obtain nutrients such as nitrogen and phosphorus that support both the coral host and if present, the Symbiodiniaceae (Fitt & Cook, 2001; Muscatine & Porter, 1977). The heterotrophic uptake of POM is facilitated by morphological adaptations such as feeding tentacles, mesenterial filaments, cnidae and even mucus, to effectively capture prey from the water column (Al‐Sofyani & Niaz, 2007; Yosef et al., 2020). The extent to which corals rely on autotrophy and heterotrophy not only varies considerably between species but is also modulated by the environment (Palardy et al., 2008).

Environmental conditions such as water flow have been shown to affect coral feeding (Chang‐Feng & Ming‐Chao, 1993; Fabricius et al., 1995; Sebens et al., 1997, 1998; Sebens & Johnson, 1991; Wijgerde et al., 2012). Corals, as sessile organisms, depend on water motion to supply food items for heterotrophic feeding. Consequently, heterotrophy in corals can be enhanced with high water flow, as there is an increased flux of food particles across the polyps (Fabricius et al., 1995). Flow speeds have also been found to influence food capture efficiency, with zooplankton primarily captured at low flow and phytoplankton captured at higher flows, allowing corals to exploit different food sources under different flow regimes (Orejas et al., 2016). However, higher water flow has variable and not always beneficial effects on nutrient acquisition, with evidence of increased uptake yet also increased efflux of nutrients (Borchardt et al., 1994). In addition, with very high water motion, the mechanical forces on the coral may become too intensive and feeding structures may be swept back (Purser et al., 2010) and possibly damaged (Sebens, 1997; Wainwright & Koehl, 1976), or particles may simply pass over polyps too quickly to be successfully captured (McFadden, 1986; Purser et al., 2010). Autotrophy can accelerate in higher flow environments, with increased oxygen efflux and thereby increased photosynthetic efficiency of the coral (Finelli et al., 2006; Mass et al., 2010). Corals have even been found in low flow conditions, to perform cilia‐induced mixing of the coral diffusive boundary layer to remove excess oxygen and prevent oxidative stress (Pacherres et al., 2022).

Whilst there is a considerable amount of literature that covers the effects of water flow on feeding regimes in corals, these studies largely focus on scleractinian corals (Orejas et al., 2016; Sebens et al., 1997, 1998; Sebens & Johnson, 1991; Wijgerde et al., 2012), with substantially less attention paid to soft corals (Chang‐Feng & Ming‐Chao, 1993; Fabricius et al., 1995). In recent years, soft corals have increased in cover in many regions, while scleractinians have concomitantly decreased and/or not recovered from bleaching events at a significant pace (Contreras‐Silva et al., 2020; Lenz et al., 2015). This is a consequence of increased environmental stressors that negatively affect scleractinian corals, in combination with soft corals' opportunistic lifestyle involving fast growth rates, extensive asexual reproduction and high fecundity (Fabricius, 1995; Haverkort‐Yeh et al., 2013; Tilot et al., 2008). One markedly successful soft coral family is Xeniidae (McFadden et al., 2023). Research on these corals has shown them to be resilient against numerous global and local change parameters. For example, a study on *Xenia* cf *crassa* (Schenk, 1896) revealed it was not vulnerable to thermal stress, with no evidence of bleaching during the marine heatwave in Australia in 2019 (Steinberg et al., 2022). In another study, *Ovabunda macrospiculata* (Gohar, 1940) demonstrated a resistance to high pco_2_ conditions (Gabay et al., 2014). One species in particular, namely *Xenia umbellata* (Lamarck, 1816), has displayed resistance to warming (Mezger et al., 2022; Thobor et al., 2022), organic eutrophication (Simancas‐Giraldo et al., 2021; Vollstedt et al., 2020) and phosphate enrichment (Klinke et al., 2022; Mezger et al., 2022), with its success across all these studies attributed to its trophic plasticity.

**FIGURE 1:**
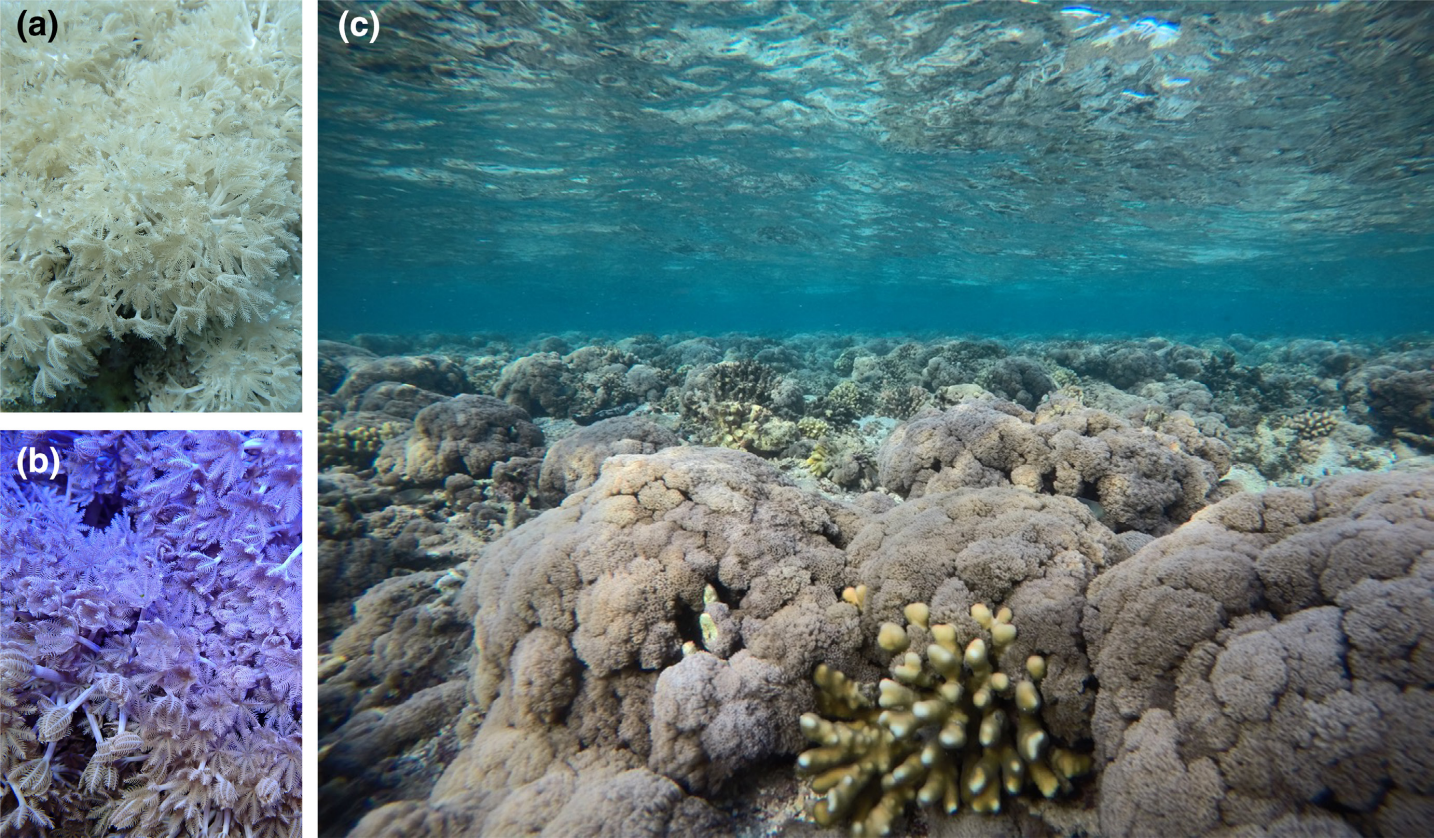
(a) *Xenia umbellata* photographed in situ; (b) *X. umbellata* photographed in laboratory conditions in the long‐term maintenance tank within the Marine Ecology department at the University of Bremen, Germany. (c) An image of a shallow reef (<1 m depth) covered in *X. umbellata* in the central Red Sea, along the coast of Saudi Arabia. Photo credit: (a, c) Walter A. Rich; (b) Arjen Tilstra.

*Xenia umbellata* is inherently mixotrophic in its feeding strategy, possessing morphological features to support both auto‐ and heterotrophy. However, whether *X. umbellata* has a preferred or more dominant feeding mode is unclear. Whilst soft corals are generally considered as more heterotrophic (Pupier et al., 2021), *X. umbellata* has demonstrated a higher photosynthetic productivity compared to other soft corals, with the ability to sustain its energetic needs by net autotrophy alone (Mezger et al., 2022). Yet, other studies suggest that *X. umbellata* relies more on heterotrophic suspension feeding because of its morphology and biochemical composition (Al‐Sofyani & Niaz, 2007). *Xenia umbellata* is naturally distributed throughout the Red Sea and the Indo‐Pacific (Verseveldt, 1965), where it occupies a range of environments including hard and soft substrates such as reef walls and sand slopes (Janes, 2014; Figure 1a,b). *Xenia umbellata* also inhabits a depth profile extending from 3 to 25 m (Janes, 2014), but has been observed at very shallow depths of less than 1 m (Figure 1c), where environmental conditions such as water motion vary considerably. Whilst water flow and its interaction with feeding is well‐researched for scleractinian corals, knowledge gaps remain for soft corals, as mentioned above. The soft coral *X. umbellata* has repeatedly demonstrated resilience in the face of global change; however, detailed knowledge about its trophic ecology is still lacking. Therefore, *X. umbellata* is the ideal soft coral to use in our investigation into the effects of water flow and feeding.

Our study aimed to (i) investigate the effects of water flow on the feeding regime of *X. umbellata* and also aimed to (ii) determine the feeding preferences of *X. umbellata*. We manipulated feeding and water flow over 15 days and assessed multiple physiological parameters including pulsation rate, growth rate, isotopic and elemental ratios as well as photo‐physiological parameters of the Symbiodiniaceae, including cell density, mitotic index and chlorophyll‐*a* content. Firstly, we hypothesised that the highest water flow treatment combined with food addition would result in the best physiological responses from *X. umbellata*, because they would be able to optimally perform both autotrophy and heterotrophy and thereby fully meet their metabolic requirements. Secondly, we hypothesised that *X. umbellata* would have a negative physiological response in the control treatment where they are unable to feed heterotrophically, and through this demonstrate their preference for a mixotrophic feeding strategy for optimal health.

## 2 METHODOLOGY

**FIGURE 2:**
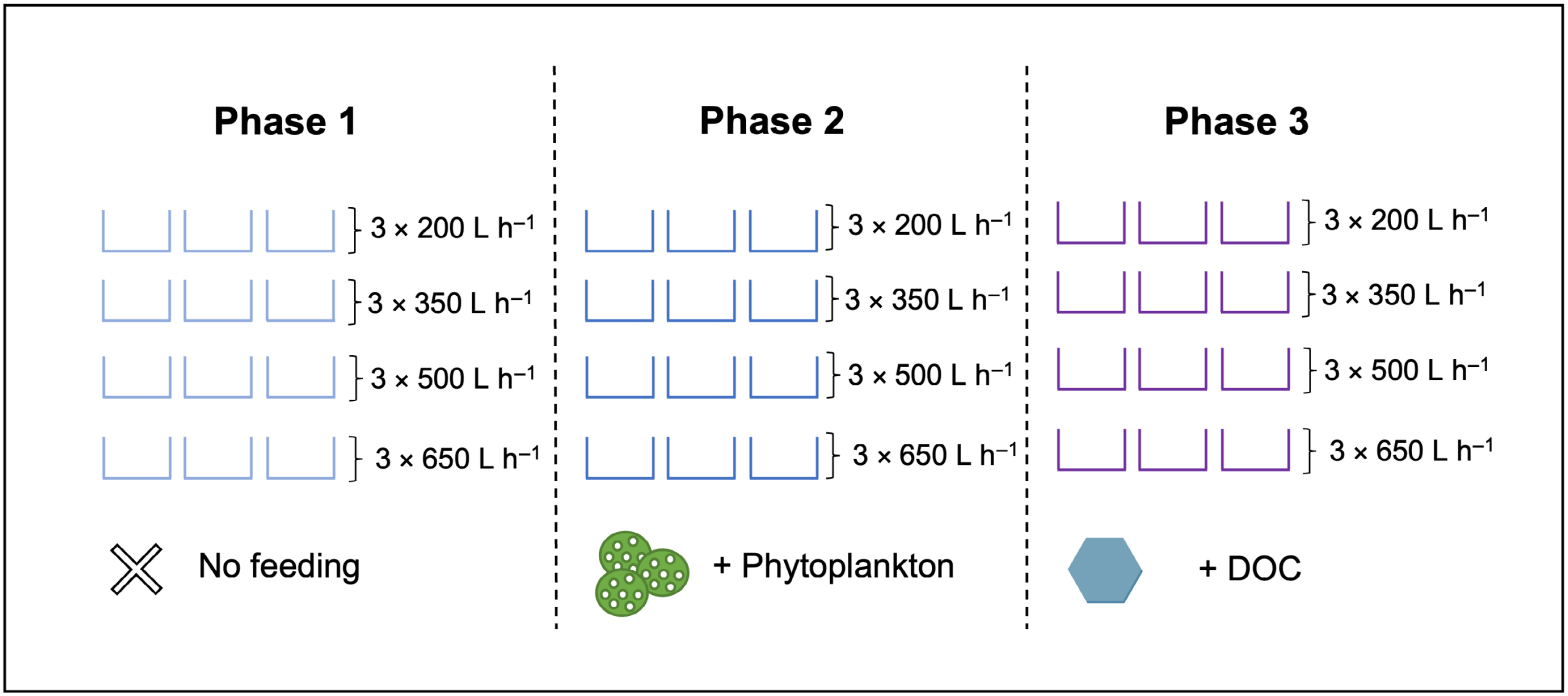
A summary of the experimental design. Feeding treatments were provided in ‘phases’, with no food supplied in Phase 1 (control), phytoplankton supplied in Phase 2 and dissolved organic carbon [DOC] supplied in Phase 3. Within each experimental phase, four water volume exchange rates (200, 350, 500 and 650 L h^−1^) were constantly maintained with a replication of three, with a random distribution across 12 experimental tanks. Phases were run consecutively for a duration of 15 days each, using different coral fragments.

The experiment was conducted within the laboratory facilities of the Marine Ecology Department at the University of Bremen, Germany, from November 2021 to February 2022. The experiment was broken down into three consecutive 15‐day phases. Four water volume exchange rates were crossed in a fully factorial design with feeding treatments supplied in phases: (i) no feeding (Phase 1), (ii) POM, supplied as phytoplankton (Phase 2) and (iii) DOC, supplied as glucose (Phase 3; Figure 2). Within each phase, the four water volume exchange rates were replicated three times, with six fragments within each tank (Figure 2).

### 2.1 *Xenia umbellata* preparation

*Xenia umbellata* colonies used in our experiment were collected in 2017 from the northern Red Sea. They were kept in a maintenance aquarium under stable temperature, salinity, light, oxygen, pH and nutrient conditions, which were later replicated in the experiment (Figure 1b; Table 1). These large clonal mother colonies of *X. umbellata*, all of the same genotype, were fragmented following the ‘plug mesh method’ outlined by Kim et al. (2022) into small fragments. The new fragments were left to heal for 14 days and acclimatise to their surroundings for a further 7 days in stable conditions, within the same maintenance aquarium as described above. Fragments were then examined for quality, and a total of 72 healthy colonies that displayed consistent and regular pulsations were selected and distributed across 12 experimental tanks, with six colonies assigned to each tank on Day 0. The fragmentation process was repeated ~21 days in advance of each experimental phase as new fragments were used within each phase. We chose to do this to ensure that corals in all phases had not been exposed to any prior stressors. In addition, this way, corals in all phases were of a similar size, removing this as a potentially confounding factor.

**TABLE 1:** Water parameters monitored throughout all three experimental phases.

	Phase 1				Phase 2				Phase 3			
	No feeding				Phytoplankton				DOC			
	200	350	500	650	200	350	500	650	200	350	500	650
Temperature (°C)	25.3 ± 0.3	25.3 ± 0.4	25.5 ± 0.2	25.3 ± 1.0	25.3 ± 0.3	25.2 ± 0.9	25.4 ± 0.2	25.2 ± 0.4	25.3 ± 0.3	25.4 ± 0.5	25.4 ± 1.0	25.4 ± 0.2
Salinity (PSU)	35.3 ± 0.3	35.2 ± 0.4	35.2 ± 0.4	34.9 ± 0.6	35.6 ± 0.5	35.4 ± 1.5	35.6 ± 0.4	35.4 ± 0.5	35.3 ± 0.5	35.2 ± 0.5	35.3 ± 0.5	35.1 ± 0.6
Oxygen (mg L^−1^)	6.5±0.087	6.5 ± 0.1	6.5 ± 0.1	6.5 ± 0.1	6.6 ± 0.1	6.7 ± 0.2	6.6 ± 0.1	6.6 ± 0.1	6.5 ± 0.1	6.5 ± 0.1	6.5 ± 0.1	6.5 ± 0.1
pH	8.3 ± 0.1	8.3 ± 0.1	8.3 ± 0.1	8.3 ± 0.1	8.3 ± 0.0	8.3 ± 0.0	8.4 ± 0.0	8.3 ± 0.0	8.3 ± 0.0	8.3 ± 0.0	8.3 ± 0.0	8.3 ± 0.0
Phosphate (mg L^−1^)^a^	<0.02	<0.02	<0.02	<0.02	<0.02	<0.02	<0.02	<0.02	<0.02	<0.02	<0.02	<0.02
Nitrate (mg L^−1^)^a^	0.5–1	0.5–1	0.5–1	0.5–1	0.5–1	0.5–1	0.5–1	0.5–1	0.5	0.5	0.5	0.5
Nitrite (mg L^−1^)	0.03 ± 0.0	0.04 ± 0.0	0.04 ± 0.0	0.04 ± 0.0	0.05 ± 0.1	0.07 ± 0.1	0.07 ± 0.1	0.09 ± 0.1	0.01 ± 0.0	0.01 ± 0.0	0.01 ± 0.0	0.01 ± 0.0
Ammonium (mg L^−1^)^a^	<0.05	<0.05	<0.05	<0.05	<0.05	<0.05	<0.05	<0.05	<0.05	<0.05	<0.05	<0.05
Calcium (mg L^−1^)	426.7 ± 17.8	421.7 ± 21.7	423.3 ± 20.6	416.7 ± 23.9	420 ± 14.77	416.7 ± 11.6	418.3 ± 18.0	418.3 ± 10.3	416.7 ± 20.6	428.3 ± 27.6	428.3 ± 23.3	421.7 ± 32.4
Magnesium (mg L^−1^)	1433.3 ± 126.6	1458.3 ± 117.7	1436.7 ± 102.3	1443.3 ± 118.7	1330 ± 63.5	1323.3 ± 63.7	1331.7 ± 50.8	1311.7 ± 57.5	1353.3 ± 156.6	1341.7 ± 94.0	1341.7 ± 175.7	1358.3 ± 32.4
Alkalinity (°dKH)	7.2 ± 1.1	7.4 ± 1.4	7.3 ± 1.2	7.4 ± 1.4	8.4 ± 0.5	8.6 ± 0.7	8.6 ± 0.7	8.5 ± 0.5	7.7 ± 1.0	7.8 ± 1.2	7.8 ± 1.1	7.6 ± 1.0

*Note*: Values are reported as averages ± standard deviation. Water flow speeds are measured in Lh^‐1^.^‐^

Abbreviation: DOC, dissolved organic carbon.

^a^ Values are displayed as ranges because data are categorical, and therefore, no average/standard deviation could be obtained.

### 2.2 Experimental setup

Each experimental tank (*n* = 12) was connected to a technical tank (*n* = 12) positioned at a lower level, behind. Each technical tank contained a skimmer (EHEIM Skim Marine 100; EHEIM GmbH and Co. KG) connected to an external air pump (EHEIM Air Compressor 100 L H^−1^, EHEIM GmbH and Co. KG), a thermostat (3613 aquarium heater. 75 W 220–240 V; EHEIM GmbH and Co. KG) connected to a temperature controller (Schego Temperature Controller TRD, max. 1000 W) and a water pump (EHEIM CompactOn 300/1000 pump; EHEIM GmbH and Co. KG). An exchange of water was constantly maintained between the two tanks via an overflow pipe and a return water pump (set to the desired water flow for the respective treatment, see Table S1). LED lights (Royal Blue—matrix module and Ultra Blue White 1:1—matrix module, WALTRON daytime® LED light) were secured above all experimental tanks and provided light on a 12:12 h light : dark cycle at an intensity of 120 μmol photons m^−2^ s^−1^ photosynthetically active radiation (PAR). Black plastic sheets were secured externally on the left and right walls and beneath every experimental tank. This ensured that all tanks were receiving the same quantity of light, regardless of their placement.

### 2.3 Water flow treatments

Within the 12 experimental tanks, four water volume exchange rates of 200, 350, 500 and 650 L h^−1^ were established (here on referred to as water flow treatments), in three replicates (see Table S1). Pumps were tested prior to the experiment for an accurate measurement of the volume exchange rate compared to the setting of the pump (see Table S1). Clod cards were used to assess and confirm the ecological significance of each speed.

### 2.4 Feeding treatments

Feeding treatments were supplied in three phases. In the first phase, no food was supplied to the corals. In the second phase, 1.5 × 10^4^ cells mL^−1^ of phytoplankton (Plankton24.de, *Synechococcus* sp.) were administered to each tank daily. This concentration was chosen because it falls within the range of conditions in the central Red Sea, where *X. umbellata* naturally occurs (Kürten et al., 2015). In the third experimental phase, DOC, in the form of glucose (D‐Glucose anhydrous, purity: 99%, Fisher Scientific U.K. Limited), was administered to each tank to achieve a constant concentration of 20 mg L^−1^ of DOC. This concentration of DOC was chosen because it was higher than ambient levels, thereby providing *X. umbellata* with ample opportunity to feed heterotrophically (Vollstedt et al., 2020). To achieve this concentration, water samples were taken on Day 0 of Phase 3 and run on a Total Organic Carbon Analyser (TOC‐L CPH/CPNPC‐Controlled Model) to determine the baseline of DOC present in each tank, and glucose was administered accordingly. Water samples were analysed as described above, for the first 3 days of the experimental phase to determine the average uptake of glucose. Consequently, 2 mg L^−1^ of glucose was administered every 2 days to all experimental tanks throughout Phase 3 of the experiment. In all three experimental phases, the skimmers were turned off for 2 h every day between 10 a.m. and 12 p.m. to provide *X. umbellata* with the opportunity to uptake the food.

### 2.5 Maintenance

In all experimental phases, salinity, temperature and oxygen were measured daily every morning in all tanks, while nutrient concentrations and pH were measured twice per week in the morning before food was supplied (Table 1). Manual adjustments were made when required. In addition, 10% water exchanges were performed daily in all experimental tanks, 2 h post‐feeding in the afternoon. In the phytoplankton feeding phase, however, 50% water exchanges were required daily due to elevated nutrient levels and increased light attenuation because of the treatment.

### 2.6 Ecological assessments

#### 2.6.1 Pulsation

One pulsation cycle was defined as one whole contraction of the polyp (open—fully closed—open; Vollstedt et al., 2020). To determine the pulsation rate, the number of pulsations displayed within 1 min was counted, with the use of a stopwatch and a hand‐tally counter. On Day 15, the pulsation rates were measured on the same three corals within each experimental tank (12 biological replicates per treatment). The measurement was focused on one random polyp per coral and was repeated three times on the same polyp (three technical replicates). To minimise variability among repeated measurements, one observer coherently performed all pulsation measurements.

#### 2.6.2 Growth rate

One colony within each experimental tank (*n* = 12) was followed throughout each 15‐day phase and counted twice each, resulting in 12 biological replicates and two technical replicates. The number of polyps on the selected colonies was counted at the beginning (Day 0) and end (Day 15) of each phase. The colony was transferred to a temporary smaller glass jar, and tweezers were used to aid and improve the accuracy of counting. To calculate the growth rate (number of new polyps per day), the equation below was used (Equation 1), where the number of polyps on Day 0 (*P*_start_) were subtracted from Day 15 (*P*_end_) and divided by the total number of days of the experimental phase *(d)*.

(1) $$\text{Growth rate}\;\left(\text{polyps}\;{\mathrm{day}}^{-1}\right)=\left(\frac{P_{\mathrm{end}-}P_{\text{start}}}{d}\right)$$

### 2.7 Symbiodiniaceae parameters

*Xenia umbellata* colonies were removed from the experiment on Day 15 and stored at −20°C. On the day of analysis, colonies were thawed for approximately 30 min in the dark. To obtain a tissue slurry, each colony was homogenised in 10 mL of demineralised (DM) water using a hand‐homogeniser (Pupier et al., 2018). An accurate sample volume was determined using a pipette. Two subsamples were created per colony, by transferring 2 mL of slurry into two Eppendorf tubes. Samples were centrifuged for 10 min to separate coral tissue and algal cells. The supernatant was discarded, and the pellet was resuspended in 2 mL of DM water. The centrifugation step was repeated once more. One subsample was used for algal cell counts and mitotic index, while the second subsample was used for chlorophyll‐*a*.

#### 2.7.1 Algal cell density and mitotic index

To obtain algal cell counts, an established counting method using a haemocytometer was followed (LeGresley & McDermott, 2010). In brief, the pellets were resuspended in 2 mL of DM water and vortexed. The haemocytometer was sterilised with ethanol and the coverslip was affixed using DM water. 10 μL of sample was pipetted beneath the coverslip onto the upper and lower counting chambers. Under a light microscope, the algal cells were counted in the four outer squares within both the upper and lower chambers, providing two replicate counts. The counts were normalised to the initial sample volume and per surface area of each colony. Cells in mitosis were also counted simultaneously and divided by the total number of algal cells per sample to obtain the mitotic index.

#### 2.7.2 Chlorophyll‐*a* per cell

For the determination of chlorophyll‐*a*, the methodology of Jeffrey and Humphrey (1975) was followed. Pellets were resuspended in 2 mL of 90% acetone and vortexed. The samples were then stored in the dark at 4°C for 24 h. After this, samples were centrifuged for 5 min, and 2 mL were transferred into two glass cuvettes (1 mL in each), for two replicate readings. Samples were individually measured at two fixed wavelengths of 663 and 630 nm using a Trilogy Fluorometer (Turner Designs) fitted with a chlorophyll‐*a* module. Each sample was measured three times. All analyses were performed in a dark room. Measurements were normalised to the initial volume of the sample and to the number of algal cells per colony.

### 2.8 Stable isotope and carbon and nitrogen elemental analyses

One colony of *X. umbellata* per treatment was removed from the experiment on Day 0, and on Day 15, rinsed with DM water to remove salt and stored at −20°C until further analysis.

Colonies were weighed and dried in the oven at 40°C for ~48 h or until a consistent weight was achieved. Dried tissue was then ground with a pestle and mortar, and ~1 mg was transferred into a tin cup. Samples were analysed for carbon and nitrogen content, as well as for isotopic ratios of δ^15^N and δ^13^C (‰) at the Natural History Museum, Berlin with a Flash 1112 EA coupled to a Delta V IRMS via a Conflow lV‐interface (Thermo Scientific), as described in greater detail in Karcher et al. (2020).

### 2.9 Statistical analyses

Firstly, we tested for normality using visual normality distribution plots and the Shapiro–Wilk normality test. Data that was not normally distributed was transformed either via a log transformation or a Tukey ladder of powers transformation. All data was first assessed for significance using a two‐way analysis of variance test (2‐ANOVA). When water flow was excluded, data were re‐assessed using a one‐way analysis of variance test (ANOVA). For significant variables, that is when *p* < .05, a post‐hoc Tukey HSD test was performed to identify specifically where the significant differences lay.

Due to different starting values of certain response parameters between experimental phases (i.e. pulsation rates, all Symbiodiniaceae parameters and all isotope and elemental parameters), statistical analyses were performed on relative differences within each experimental phase. However, we opted to show absolute values within each figure for transparency and easier understanding. Consequently, statistically significant differences between Day 15 data marked on the figures may not visually appear as very different. Figures with visualised relative differences can be found in the Appendix S1 (see Figures S1, S3, and S4).

All data analyses and creation of figures were carried out in R (version 4.2.3; R Core Team, 2023) using packages ‘dplyr’ (Wickham et al., 2018), ‘ggplot2’ (Wickham, 2016), ‘ggpubr’ (Kassambara, 2020a), ‘RColorBrewer’ (Neuwirth, 2014), ‘wesanderson’ (Ram & Wickham, 2018), ‘gridExtra’ (Auguie & Antonov, 2017), ‘rstatix’ (Kassambara, 2020b), ‘ARTools’ (Kay et al., 2021) and ‘rcompanion’ (Mangiafico, 2017).

## 3 RESULTS

### 3.1 Water flow

Water flow had no observable nor significant effect on any of the measured physiological parameters of *X. umbellata* (see Figures S1–S4; Table S2). Consequently, data from the water flow treatments have been pooled and only feeding treatments are presented within the figures below, with a replication of *n* = 12 per feeding treatment on Day 15. On Day 0, however, no data could be pooled as on Day 0 no water flow treatments were established yet. Therefore, the replication number presented within the results section for Day 0 remained as three.

### 3.2 Pulsation

**FIGURE 3:**
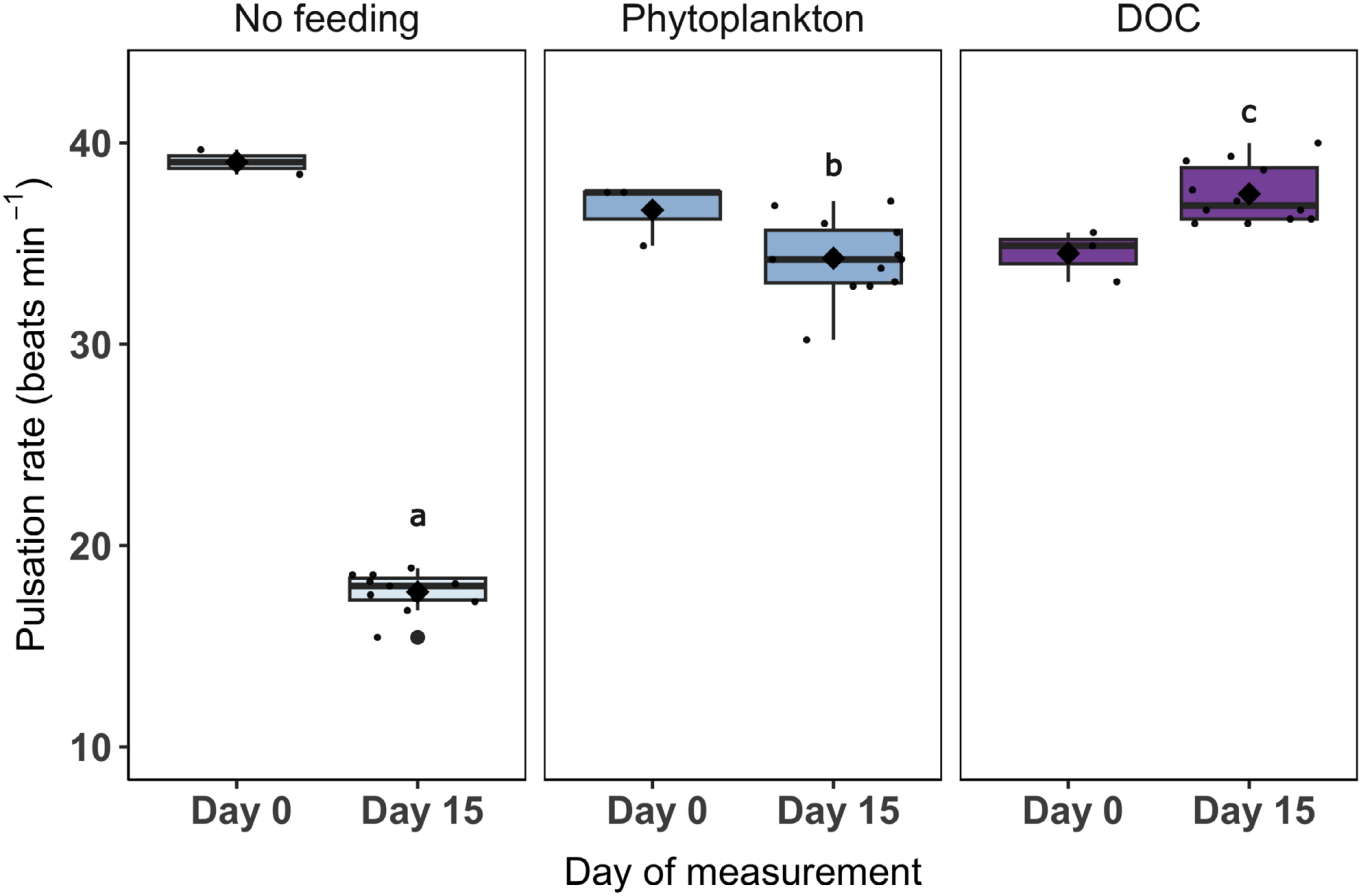
The average pulsation rate (beats min^−1^) of *Xenia umbellata* at the start (Day 0) and end (Day 15) of the experiment within ‘No feeding’, ‘Phytoplankton’ and ‘Dissolved organic carbon (DOC)’ feeding treatments. On Day 0, there are three biological replicates for each feeding treatment, and on Day 15, there are 12 biological replicates. The median is represented by the black horizontal line and the mean is indicated by a black diamond. Variables that have different letters are significantly different (based on relative differences between Day 15 data, see Section 2.9 **)**, whereas variables that have the same letter are not significantly different.

Feeding treatments had a significant effect on pulsation rates (ANOVA, 2, *F* = 905.2, *p* < .001). Post‐hoc testing revealed that all three feeding treatments were significantly different (Tukey, *p* < .001; Figure 3). On Day 15, pulsation rates were significantly lowest in the no‐feeding treatment, where pulsations had decreased by a magnitude of 1.9, from 39 down to 21 beats min^−1^. Where food was provided, in the phytoplankton and DOC feeding treatments, the pulsation rate remained more stable, with a marginal decrease from 36 beats min^−1^ to 34 beats min^−1^ in the phytoplankton treatment and with a marginal increase from 34 to 37 beats min^−1^ in the DOC treatment (Figure 3). On Day 15, corals in the DOC treatment had a significantly higher pulsation rate than all other treatments (Tukey, *p* < .001; Figure 3).

### 3.3 Growth rate

**FIGURE 4:**
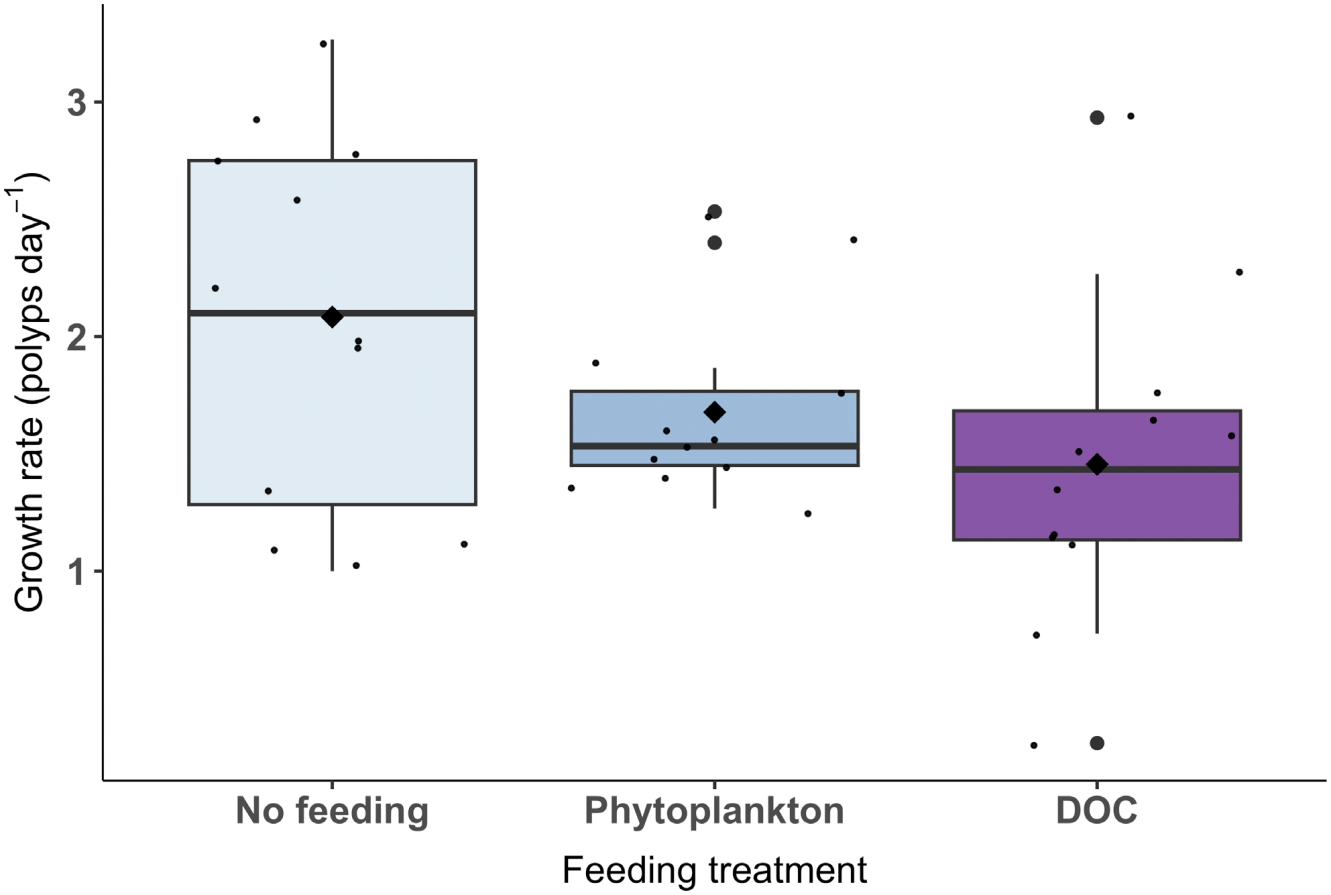
The average growth rate of *Xenia umbellata* following 15 days of exposure to ‘No feeding’ ‘Phytoplankton’ and ‘Dissolved organic carbon (DOC)’ feeding treatments. There are 12 biological replicates per feeding treatment. The median is represented by the black horizontal line and the mean is indicated by a large black diamond.

Growth rates were highly variable among treatments, with an average growth of 2.1 polyps per day in the no‐feeding treatment, 1.7 polyps per day in the phytoplankton treatment and 1.5 polyps per day in the DOC treatment (Figure 4). These were not significantly different (ANOVA, 2, *F* = 2.848, *p* = .07).

### 3.4 Symbiodiniaceae parameters

See Figure 5.

**FIGURE 5:**
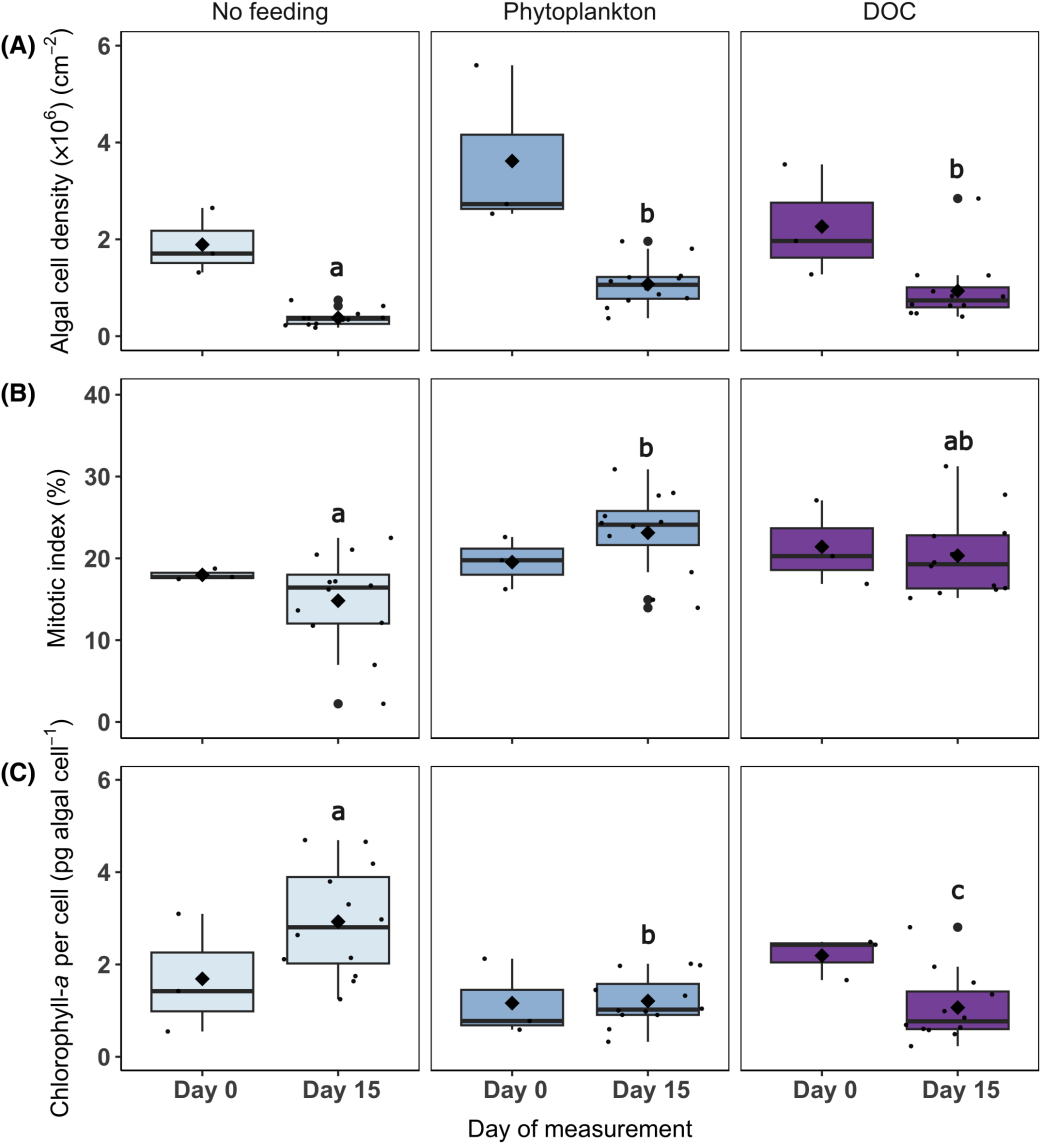
Symbiodiniaceae parameters of *Xenia umbellata*, including (A) algal cell density (cm^−2^); (B) mitotic index (%); and (C) chlorophyll‐*a* per algal cell (pg algal cell^−1^) at the start (Day 0) and end (Day 15) of the experiment within ‘No feeding’, ‘Phytoplankton’ and ‘Dissolved organic carbon (DOC)’ feeding treatments. Day 0 for all Symbiodiniaceae parameters has a biological replication of three, and Day 15 has a biological replication of 12 per feeding treatment. The median is represented by the black horizontal line and the mean is indicated by a large black diamond. Variables that have different letters are significantly different, whereas variables that have the same letter are not significantly different (based on relative differences between Day 15 data, see Section 2.9 **).**

### 3.5 Algal cell density

Significant differences in algal cell density were observed across feeding treatments (ANOVA, 2, *F* = 15.87, *p* < .001; Figure 5A). Whilst the algal cell density decreased throughout the experiment in all treatments, there was a greater drop in the no‐feeding treatment, where the algal cell density was significantly lower (3.8 × 10^5^ cells cm^−2^) than in both the phytoplankton (1.1 × 10^6^ cells cm^−2^) and DOC (9.4 × 10^5^ cells cm^−2^) feeding treatments on Day 15 (Tukey, *p* < .01; Figure 5A). Significantly higher algal cell densities were observed when food was supplied as phytoplankton (Tukey, *p* < .01) and as DOC (Tukey, *p* < .001) compared to the no‐feeding treatment (Figure 5A).

### 3.6 Mitotic index

Overall, there was a significant effect of feeding treatments on the mitotic index of the corals (ANOVA, 2, *F* = 5.144, *p* < .05), with a significantly higher number of cells in mitosis in the phytoplankton treatment (23%) compared to the no‐feeding treatment (15%) on Day 15 (Tukey, *p* < .05; Figure 5B). There was no significant difference between no feeding and DOC (Tukey, *p* > .05) and between phytoplankton and DOC (Tukey, *p* > .05; Figure 5B).

### 3.7 Chlorophyll‐*a*

Feeding treatments had a strong significant effect on the chlorophyll‐*a* content per cell of *X. umbellata* (ANOVA, 2, *F* = 16.65, *p* < .001), with all treatments differing significantly (Tukey, *p* < .05; Figure 5C). A significantly higher chlorophyll‐*a* per cell was found in the no‐feeding treatment (2.9 pg cell^−1^) compared to both the phytoplankton (1.2 pg cell^−1^) and DOC treatments (1.1 pg cell^−1^; Figure 5C). In addition, the chlorophyll‐*a* per cell was significantly higher in the phytoplankton treatment compared to the DOC (Tukey, *p* < .05; Figure 5C).

### 3.8 Isotope and elemental analyses

**FIGURE 6:**
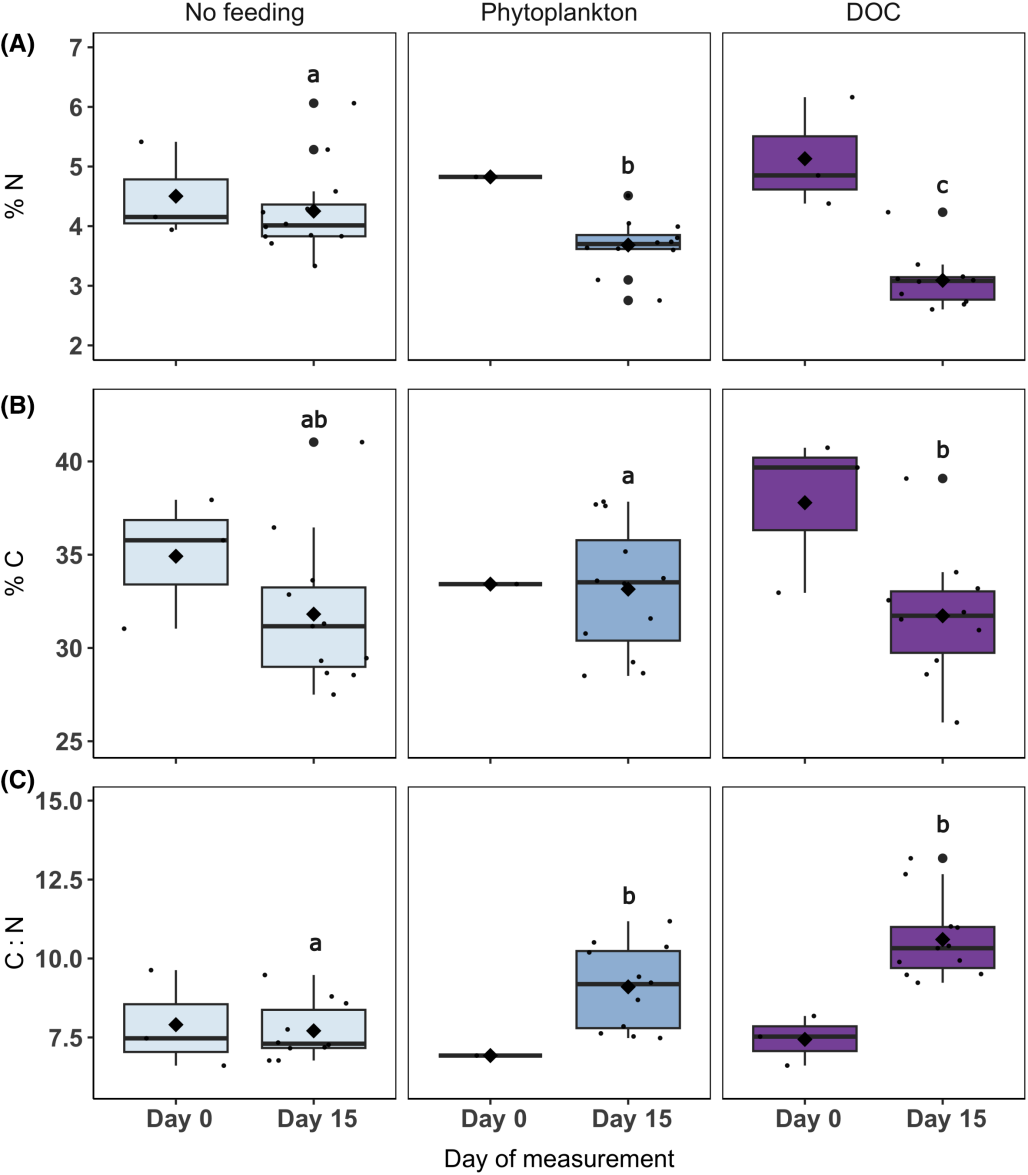
Elemental data of *Xenia umbellata* including (A) nitrogen content (%N), (B) carbon content (%C) and (C) carbon‐to‐nitrogen ratio (C : N), following 15 days of exposure to ‘No feeding’ ‘Phytoplankton’ and ‘Dissolved organic carbon (DOC)’ feeding treatments. For Day 0 measurements, there are three biological replicates, excluding the phytoplankton treatment where it is reduced to 1, due to technical faults during analysis. For Day 15 measurements there are 12 biological replicates. The median is represented by the black horizontal line and the mean is indicated by a large black diamond. Statistical significance is indicated by letters. Variables that have different letters are significantly different, whereas variables that have the same letter are not significantly different (based on relative differences between Day 15 data, see Section 2.9 **).**

Significant differences in nitrogen content (%N) were observed across feeding treatments (ANOVA, 2, *F* = 12.72, *p* < .001; Figure 6A). A significantly higher nitrogen content was found in corals within the no‐feeding treatment (4.3%), compared to phytoplankton feeding (3.7%) and compared to DOC (3%; Tukey, *p* < .001; Figure 6A). In addition, the nitrogen content of corals within the phytoplankton treatment was significantly higher than corals in the DOC feeding treatment (3%; Tukey, *p* < .05; Figure 6A).

Significant differences in carbon content (%C) were also observed across feeding treatments (ANOVA, 2, *F* = 7.049, *p* < .05; Figure 6B). A significantly higher carbon content was found in corals within the phytoplankton treatment (33%) compared to the DOC treatment (31%; Tukey, *p* < .05; Figure 6B). However, no significant differences were found between corals in no‐feeding treatment (32%) compared to both food provision treatments (Tukey, *p* > .05; Figure 6B).

The carbon‐to‐nitrogen ratio (C : N) significantly differed across feeding treatments (ANOVA, 2, *F* = 21.7, *p* < .001; Figure 6C). The C : N of corals within the phytoplankton (9.1) and DOC (10.5) feeding treatments was significantly higher than that of corals in the no‐feeding treatment (7.5; Tukey, *p* > .01; Figure 6C). However, there was no significant difference in the C : N of corals between phytoplankton and DOC treatments (Tukey, *p* > .05; Figure 6C).

Lastly, no observable, nor statistically significant differences were found between feeding treatments in the nitrogen (δ^15^N) and carbon (δ^13^C) stable isotope signatures of *X. umbellata* (see Figure S5A,B).

## 4 DISCUSSION

Overall, we argue that the physiology of *X. umbellata* is unaffected by water flow as no significant effect on any of the observed parameters was found within our study. *Xenia umbellata* was, however, substantially impacted by a lack of heterotrophic food sources, with a significantly reduced pulsation rate, lower Symbiodiniaceae cell density and lower mitotic index compared to the fed treatments, yet significantly higher chlorophyll‐*a* per cell and N content. Significant differences were also observed between the DOC and phytoplankton treatments, with significantly higher pulsation rates and lower chlorophyll‐*a* per cell in the DOC treatment, but higher C and N content in the phytoplankton treatment.

### 4.1 Does water flow affect the physiology or trophic ecology of *X. umbellata*?

One key outcome of our study was finding no significant effect of water flow on any of the measured physiological parameters of *X. umbellata* (see Figures S1–S4; Table S2). Based on previous research about the effects of water flow on feeding regimes in corals, we hypothesised that there would be a positive effect on the physiological responses of *X. umbellata* due to water flow enhancing autotrophy and heterotrophy. These studies, however, have mainly focused on scleractinian corals, or soft corals with distinctly different morphologies to *X. umbellata*. The morphology of *X. umbellata* (and some other species within the Xeniidae family) is unique in that it exhibits continuous non‐synchronous pulsation of its polyps, first noted by Lamarck, 1816. Pulsation motions continually thrust water in an upward direction around the polyp, prompting mixing across the coral‐water boundary layer (Kremien et al., 2013) and thereby modulating flow at a local scale around the polyps. Consequently, we suggest that *X. umbellata* does not gain any additional benefit from a high‐flow environment, nor experience negative effects from a low‐flow environment because it is able to control flow already at a local scale, via its pulsation behaviour. It should be noted, however, that higher water flows than measured in our experiment may occasionally occur, especially in more turbulent shallow water environments that *X. umbellata* occupy. In such environments it is possible that polyps may be blasted and, therefore, unable to function, thereby making heterotrophic feeding difficult (Purser et al., 2010). We would, therefore, suggest further experimentation to look into the effects of a more extreme environment, to see whether or not *X. umbellata* still remain unaffected.

### 4.2 Does *X. umbellata* exhibit a dominant feeding mode?

Whilst water flow treatments had no significant effect on the assessed physiological parameters, we did observe significant effects of feeding treatments on *X. umbellata*.

Firstly, we found that pulsation rates were significantly different between all feeding treatments (Figure 3). Corals in the unfed treatment experienced a large reduction in pulsation rate following 15 days of no food, whereas comparatively, the pulsation rate of the corals in both the fed treatments did not differ substantially from the start of their respective Day 0 measurement (Figure 3). Given that pulsation can enhance autotrophy (Kremien et al., 2013), we expected to see increased pulsation rates in the no‐feeding treatment. However, the significantly lower pulsation rate among unfed corals suggests that *X. umbellata* may not have had the energy to sustain its normal range of pulsations nor increase its pulsation rate to enhance autotrophy when lacking a heterotrophic energy source. For example, 12 of the most common symbiotic soft coral genera on the Great Barrier Reef are unable to satisfy their carbon requirements exclusively via autotrophy (Fabricius et al., 1995). In addition, it could be that the reduced pulsation rate was intentional by the coral to conserve energy, as a result of low capture success. However, if this was the case, we would also expect to see a reduction in growth rate, yet growth was not negatively impacted and instead increased across all treatments. We would, therefore, encourage a follow‐up study where we correlate pulsation rates with the capture rate of prey to address this hypothesis better.

Although our results suggest that autotrophy was not sufficient to satisfy the energetic needs of *X. umbellata* alone, it is likely that with greater light availability, a higher or sole reliance on autotrophy may have been possible. In our experiment, we supplied a light intensity of 120 μmol photons m^−2^ s^−1^ photosynthetically active radiation (PAR). However, much higher light intensities have been recorded in the Red Sea at depths of 1–20 m than supplied in our experiment (Haas et al., 2010). For example, in the winter months light intensity can range from 78 μmol quanta m^−2^ s^−1^ (20 m) to 527 μmol quanta m^−2^ s^−1^ (1 m) and even reach 144 μmol quanta m^−2^ s^−1^ (20 m) to 946 μmol quanta m^−2^ s^−1^ (1 m) in the peak of summer (Haas et al., 2010). Although introducing food sources can increase nutrient loading and consequently decrease light availability, this did not occur here as nutrients were consistent across treatments (Table 1), and light was measured steadily at 120 μmol photons m^−2^ s^−1^ photosynthetically active radiation (PAR) across all treatments. With *X. umbellata* inhabiting depths as shallow as 1 m (Figure 1c), the light intensity in our experiment may not have been sufficiently high to support autotrophy as a sole feeding mode.

Secondly, unfed corals also had a significantly lower algal cell density compared to both food provision treatments (Figure 5A), and a significantly lower mitotic index compared to the POM feeding treatment (Figure 5B). Generally, higher algal cell densities and mitotic indices are indications of a healthy coral that has a proliferating and stable supply of symbionts (Belda et al., 1993). However, there is also evidence to suggest that higher values occur under heat stress in the hard coral *Stylophora pistillata* and could be a sign of altered resource partitioning (Rädecker et al., 2021). In this instance, however, heat stress was not a factor, and therefore, we argue that the significantly reduced algal cell density demonstrates a reduction in health of *X. umbellata* in the absence of a heterotrophic food source.

We did, however, observe a significantly higher chlorophyll‐*a* content (Figure 5C), higher nitrogen content (Figure 6A) and lower carbon‐to‐nitrogen ratio (Figure 6C) among unfed corals, compared to those in phytoplankton and DOC treatments. This increase in cellular chlorophyll‐*a* may have been the holobionts' attempt to optimise its photosynthetic capacity given that autotrophy was the only mode of energy acquisition available in the absence of heterotrophic food. These cellular morphological modifications have been observed previously in corals transplanted from deep to shallow water (Martinez et al., 2020), where autotrophy also became the primary feeding mode, and additionally in other instances where corals have experienced unfavourable conditions for autotrophy and thus needed to optimise light capture (Wall et al., 2020). The higher nitrogen content in unfed corals could, therefore, be justified by this concomitant increase in chlorophyll‐*a*, as chlorophyll‐*a* compounds contain nitrogen (Imsande, 1998).

Overall, our data suggests that *X. umbellata* may prefer the presence of a heterotrophic food source in order to maintain optimum health, thereby supporting the notion that it is an inherent mixotroph. Whilst the health of *X. umbellata* appeared to decline when in absence of food, we cannot distinctly say that they are unable to sustain themselves with autotrophy alone because the light intensity supplied within our experiment was at the lower end of their natural range. Therefore, we recommend for future work to repeat our experiment using a range of higher light intensities to thoroughly assess the role of autotrophy for *X. umbellata*, with inclusion of a photosynthesis‐irradiance (PI) curve to provide further insight.

### 4.3 Does *X. umbellata* have a preferred heterotrophic food source?

The pulsation rate between phytoplankton and DOC feeding treatments differed with a significantly higher pulsation rate among corals exposed to DOC (Figure 3). Pulsation is particularly beneficial for the uptake of dissolved matter from the surrounding water (Kremien et al., 2013), therefore justifying the increased pulsation rates observed in the DOC feeding treatment. Furthermore, corals exposed to DOC had a significantly lower concentration of chlorophyll‐*a* per algal cell compared to corals in the phytoplankton treatment (Figure 5C). This could be because experimentally provided DOC as an available carbon source leads to an excess supply of inorganic carbon, and therefore, *X. umbellata* may no longer invest energy into enhancing its photosynthetic apparatus, resulting in lower concentrations of chlorophyll‐*a*. A similar ecophysiological response was recently observed among the upside‐down jellyfish *Cassiopea* sp. where gross photosynthesis was reduced in response to medium (20 mg L^−1^) and high (40 mg L^−1^) concentrations of DOC (Tilstra et al., 2022). Lastly, a significantly higher nitrogen (%N) and carbon content (%C) was observed within the phytoplankton feeding treatment compared to the DOC feeding treatment (Figure 6A,B). It, therefore, appears that phytoplankton could serve as a more nutritious food source for *X. umbellata* as digested plankton offers a source of carbon but also organic nitrogen (Ferrier‐Pagès et al., 2003) that supports coral growth.

Our study suggests that POM provision in the form of phytoplankton, best supported the health of *X. umbellata* compared to DOM. It is important to highlight, however, that we only provided one type of DOM in the form of DOC, and one type of POM in the form of one species of phytoplankton, excluding other important groups such as dissolved inorganic nitrogen (DIN), dissolved organic nitrogen (DON) and other particulate matter such as zooplankton and additional phytoplankton species. Our experiment aimed to determine the trophic preferences of *X. umbellata*, and now upon forming a baseline understanding, future research could build upon this further by assessing a wider range of heterotrophic food sources.

## 5 CONCLUSIONS

Our study shows that *X. umbellata* is unaffected by water flow and does not gain any additional benefit from high flow nor suffers under low flow regimes. We attribute this to its ability to control water flow at a local scale around its polyps using its pulsation behaviour to continually achieve optimum flow conditions. In addition, we found that *X. umbellata* does not respond particularly well to an absence of heterotrophic food sources with significantly reduced pulsation, algal cell density and mitotic index. However, our findings suggest that photosynthetic energy generation of the *X. umbellata* holobiont is enhanced via increased chlorophyll‐*a* contents per cell when food is scarce. Lastly, we found that the health of *X. umbellata* may be better supported by carbon and nitrogen‐containing‐POM over carbon‐exclusive‐DOM ingestion, as a greater variety of nutrients are offered. Overall, our study demonstrates the flexibility of *X. umbellata* to a dynamic environment, especially those with variable water flow and food availability.

### 5.1 Ecological implications

Coastal environments are experiencing more eutrophication as a consequence of nutrient loading, with increased levels of dissolved inorganic nitrogen (DIN) and dissolved inorganic phosphate (DIP; Zhao et al., 2021). These higher nutrient levels stimulate excessive phytoplankton growth and an increase in algal biomass (Yunev et al., 2007), which, in turn, increases the release of DOC into the water (Mueller et al., 2016). Overall, these increases may favour the success of *X. umbellata* by supporting their heterotrophic feeding, whereas scleractinian corals could be negatively impacted. For example, increased DIN and DIP could cause a reduction in calcification up to 50% (Fabricius, 2005), and increased DOC could cause bleaching (Pogoreutz et al., 2017). Overall, our findings contribute toward a better understanding of how *X. umbellata* is able to occupy such a broad range of habitats with varying environmental conditions, as well as to succeed in the face of global and local change.

## AUTHOR CONTRIBUTIONS

**C.E.L. Hill:** Data curation (equal); formal analysis (lead); investigation (lead); methodology (equal); supervision (equal); validation (equal); visualization (lead); writing – original draft (lead); writing – review and editing (lead). **S.G. Abbass:** Data curation (equal); investigation (equal); methodology (equal); supervision (equal); writing – review and editing (equal). **G. Caporale:** Data curation (equal); investigation (equal); methodology (equal); writing – review and editing (equal). **Y.C. El‐Khaled:** Methodology (equal); validation (equal); writing – review and editing (equal). **L. Kuhn:** Data curation (equal); investigation (equal); methodology (equal); writing – review and editing (equal). **T. Schlenzig:** Data curation (equal); investigation (equal); methodology (equal); writing – review and editing (equal). **C. Wild:** Conceptualization (supporting); funding acquisition (lead); project administration (lead); resources (lead); writing – review and editing (equal). **A. Tilstra:** Conceptualization (lead); project administration (equal); supervision (lead); writing – review and editing (equal).

## CONFLICT OF INTEREST STATEMENT

The authors declare no competing interests.

## Supporting information


Appendix S1

## Data Availability

Data are publicly available via the online Dryad repository: 10.5061/dryad.8kprr4xtf.
