# Supplementary material for: Physiology of the widespread pulsating soft coral Xenia umbellata is affected by food sources, but not by water flow
Source: Ecol Evol. 2023 Sep 1;13(9):e10483. doi: 10.1002/ece3.10483 (PMC10472534; doi:10.1002/ece3.10483)
Supplement: Supplementary file 1 — Appendix S1 [file ECE3-13-e10483-s001.docx]

**Appendix**

Table S1 Water flow treatment details. Pumps remained consistently on these settings throughout all three experimental phases. The measured pumping speed average ± error.

| Water flow treatment | Pump model | Pump setting | Measured pumping speed (Lh^-1^) |
| --- | --- | --- | --- |
| 1 | EHEIM Compact On 300 pump GmbH and Co. KG, Germany | 200 | 205.68 ± 1.62 |
| 2 | EHEIM Compact On 1000 pump; GmbH and Co. KG, Germany | 350 | 335.26 ± 2.13 |
| 3 | EHEIM Compact On 1000 pump; GmbH and Co. KG, Germany | 500 | 493.81 ± 4.44 |
| 4 | EHEIM Compact On 1000 pump; GmbH and Co. KG, Germany | 650 | 649.93 ± 16.21 |

**
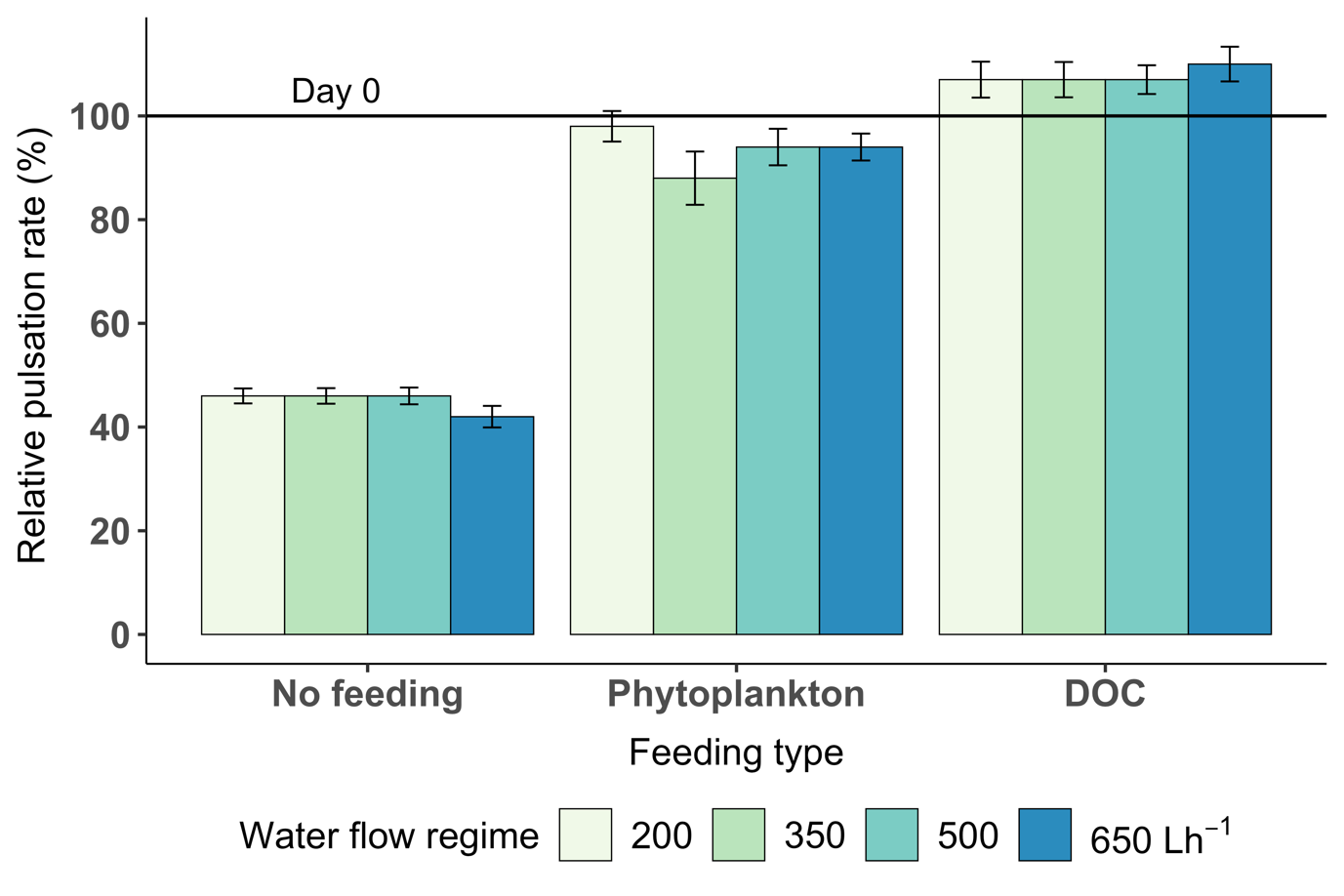
**

Figure S1 The average relative pulsation rates of Xenia umbellata on day 15, following exposure to ‘no feeding’ ‘phytoplankton’ and ‘dissolved organic carbon [DOC]’ feeding treatments crossed with four water flow speeds of 200 L h^-1^, 350 L h^-1^, 500 L h^-1^ and 650 L h^-1^. Values are reported as a relative comparison to day 0 of the respective feeding treatment. The average number of pulsations on day 0 of each feeding treatment is equivalent to 100% and is indicated by the horizontal line labelled ‘Day 0’. There was no significant effect of water flow. For significant effects of feeding treatments, please see “Results”.


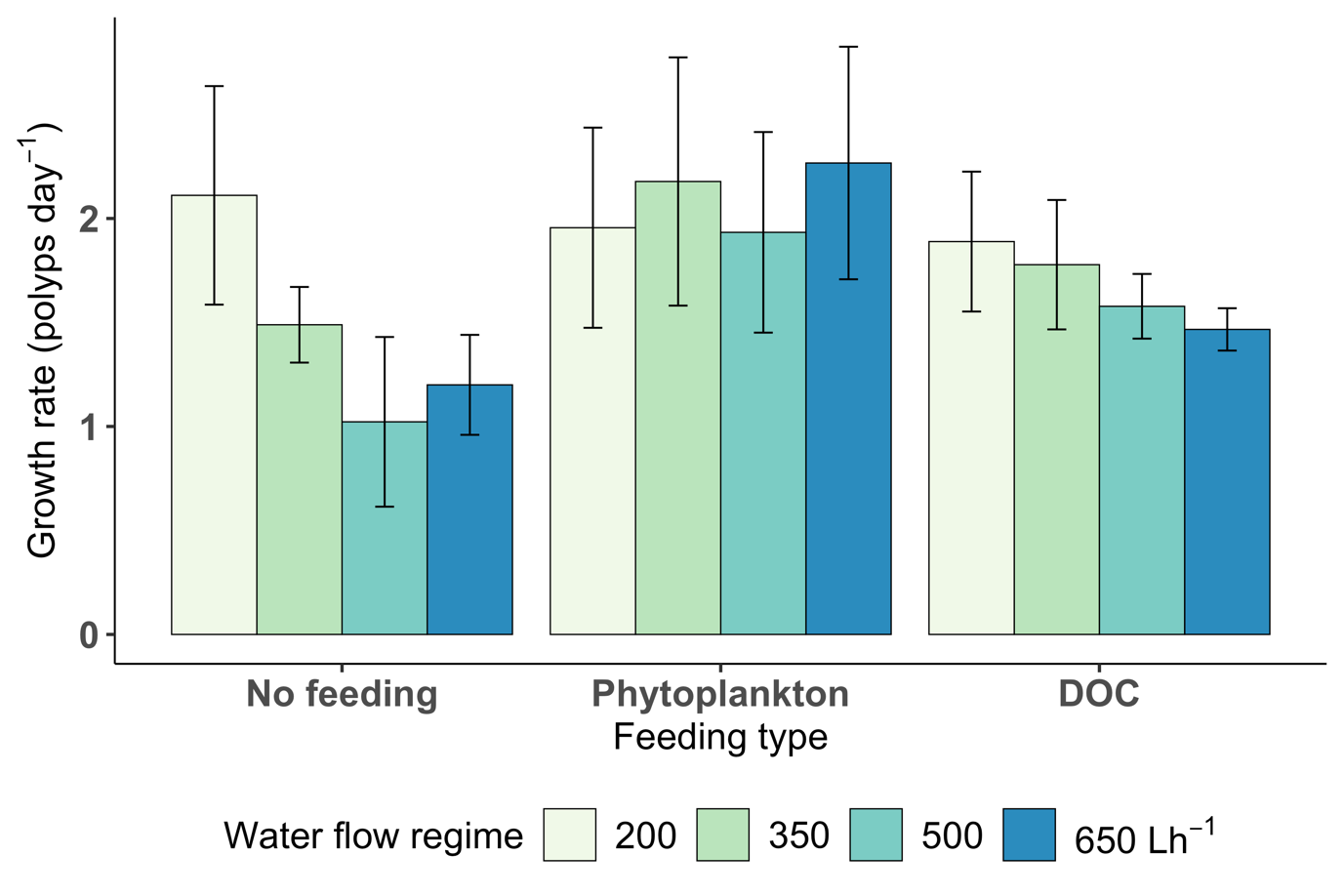


Figure S2 The average growth rate of Xenia umbellata following 15 days of exposure to ‘no feeding’ ‘phytoplankton’ and ‘dissolved organic carbon [DOC]’ feeding treatments crossed with four water flow speeds of 200 L h^-1^, 350 L h^-1^, 500 L h^-1^ and 650 L h^-1^. There was no significant effect of water flow nor feeding treatments.


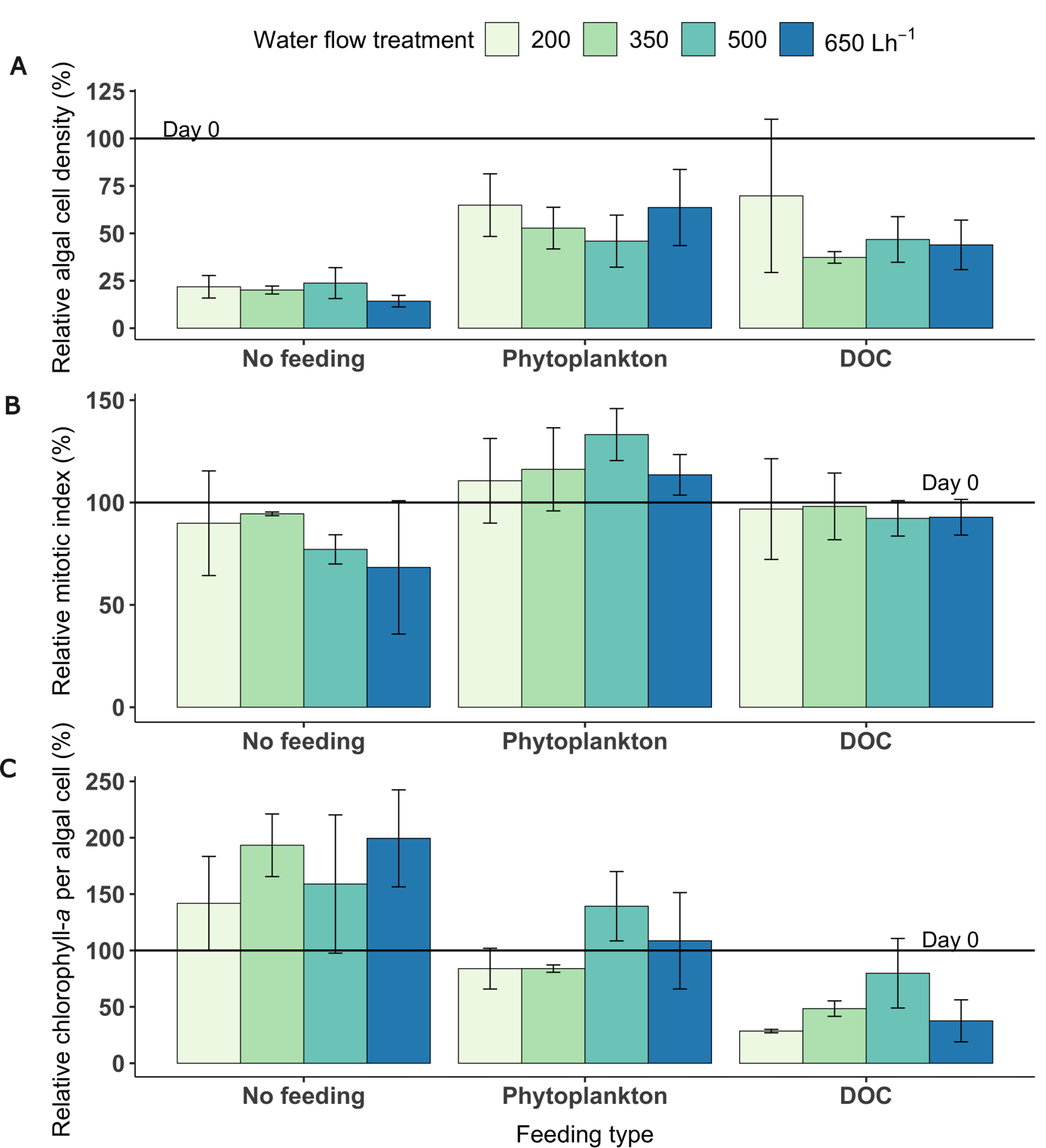


Figure S3 Relative Symbiodiniaceae parameters of Xenia umbellata including A] algal cell density (%), B] mitotic index (%) and C] chlorophyll-a per algal cell (%), following 15 days of exposure to ‘no feeding’ ‘phytoplankton’ and ‘dissolved organic carbon [DOC]’ crossed with four water flow speeds of 200 L h^-1^, 350 L h^-1^, 500 L h^-1^ and 650 L h^-1^. Values are reported as a relative comparison to day 0 of the respective feeding treatment. The average value on day 0 of each experimental phase is equivalent to 100% and is indicated by the horizontal line labelled ‘Day 0’. There was no significant effect of water flow. For significant effects of feeding treatments, please see “Results”.


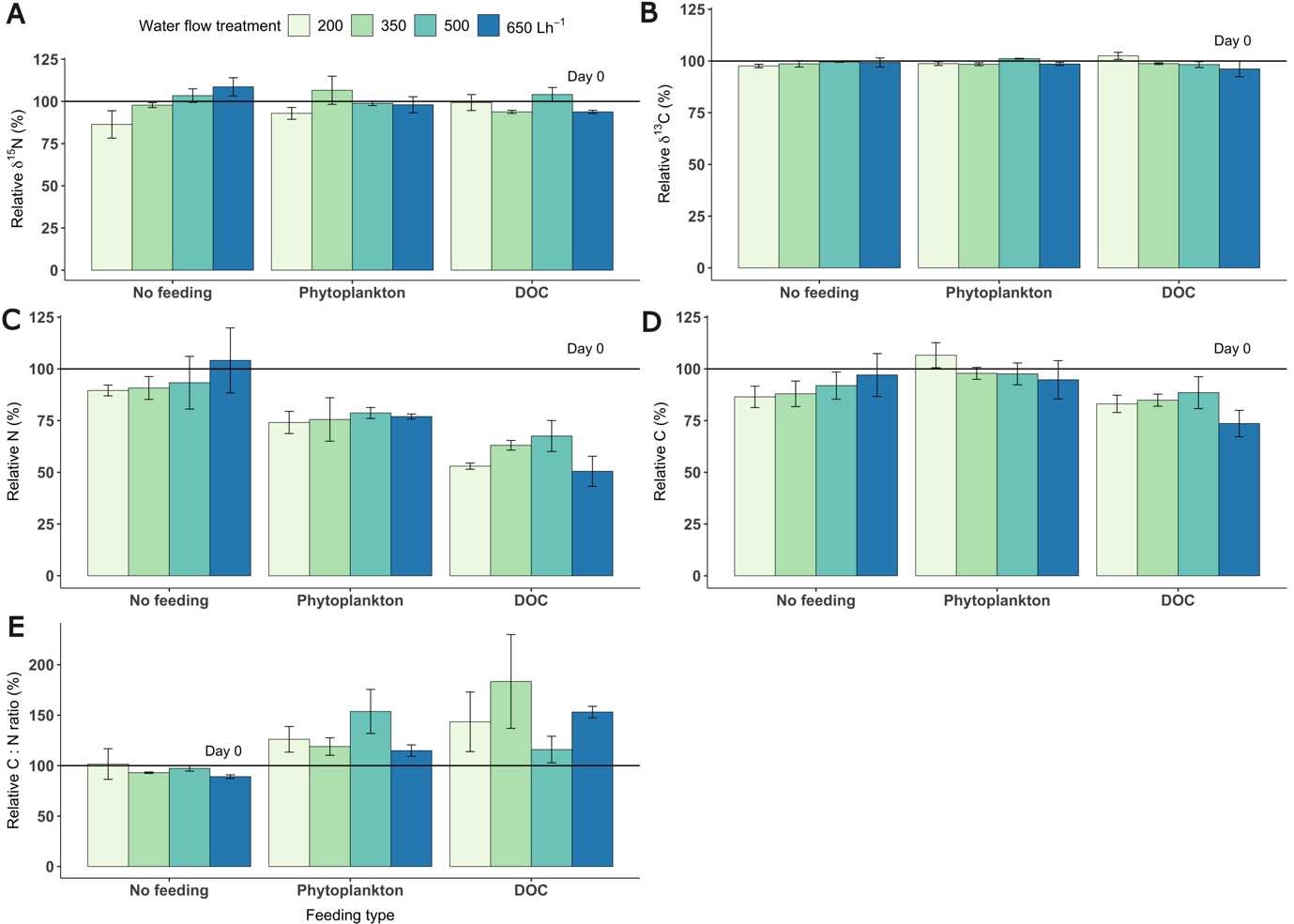


Figure S4 Relative isotope and elemental data of Xenia umbellata including A] relative nitrogen stable isotope ratio (δ^15^N (‰)) (%), B] relative carbon stable isotope ratio (δ^13^C (‰)) (%) C] relative nitrogen content (%N) (%), D] relative carbon content (%C) (%) and E] relative carbon to nitrogen ratio (C:N) (%), following 15 days of exposure to ‘no feeding’ ‘phytoplankton’ and ‘dissolved organic carbon [DOC]’ crossed with four water flow speeds of 200 L h^-1^, 350 L h^-1^, 500 L h^-1^ and 650 L h^-1^. Values are reported as a relative comparison to day 0 of the respective feeding treatment. The average value on day 0 of each experimental phase is equivalent to 100% and is indicated by the horizontal line labelled ‘Day 0’. There was no significant effect of water flow. For significant effects of feeding treatments, please see “Results”.

Table S2 A summary of the statistical output when data was assessed according to water flow and feeding with a 2-way-ANOVA. Significance is indicated by a p value < 0.05 and asterisks indicate the strength of the significance e.g., *** meaning p < 0.0001, ** meaning p < 0.01.

†Whilst water flow appeared to have a significant effect on pulsation, post hoc testing revealed no significant differences between water flow speeds within feeding treatments.

| Approach 1: Water flow and feeding | | | | | |
| --- | --- | --- | --- | --- | --- |
| Statistical test | Parameters | Term | DF | F value | P value |
| 2-way-ANOVA | Pulsation | Water flow  Feeding  Water flow * feeding | 3  2  6 | 14.947  1011.932  5.422 | 4.84 x 10^-9^ ***†  < 2 x 10^-16^ ***  2.57 x 10^-5^ ***† |
| 2-way-ANOVA | Growth Rate | Water flow  Feeding  Water flow * feeding | 3  2  6 | 0.799  2.546  0.516 | 0.5065  0.0994  0.7905 |
| 2-way-ANOVA | Symbiodiniaceae density | Water flow  Feeding  Water flow * feeding | 3  2  6 | 0.263  8.414  0.353 | 0.8515  0.0017 **  0.9008 |
| 2-way-ANOVA | Mitotic index | Water flow  Feeding  Water flow * feeding | 3  2  6 | 0.202  4.206  0.279 | 0.8939  0.0272  0.9411 |
| 2-way-ANOVA | Chlorophyll-*a* per cell | Water flow  Feeding  Water flow * feeding | 3  2  6 | 1.26  15.56  0.73 | 0.31  4.7 x 10^-5^ ***  0.63 |
| 2-way-ANOVA | Isotope δ N15 | Water flow  Feeding  Water flow * feeding | 3  2  6 | 2.170  0.730  2.102 | 0.1191  0.4929  0.0924 |
| 2-way-ANOVA | Isotope δ C13 | Water flow  Feeding  Water flow * feeding | 3  2  6 | 0.763  0.782  1.479 | 0.527  0.470  0.231 |
| 2-way-ANOVA | Element N % | Water flow  Feeding  Water flow * feeding | 3  2  6 | 0.563  11.457  0.712 | 0.644957  0.000353 ***  0.643383 |
| 2-way-ANOVA | Element C % | Water flow  Feeding  Water flow * feeding | 3  2  6 | 0.275  0.807  0.924 | 0.843  0.459  0.497 |
| 2-way-ANOVA | C : N | Water flow  Feeding  Water flow * feeding | 3  2  6 | 0.723  24.101  1.926 | 0.549  3.65 x 10^-6^ ***  0.124 |

Table S3 A summary of the statistical output when data was assessed according to feeding with a 1-way-ANOVA. Significance is indicated by a p value < 0.05 and asterisks indicate the strength of the significance e.g., *** meaning p < 0.0001, * meaning p < 0.05.

| Approach 2: Feeding | | | | | |
| --- | --- | --- | --- | --- | --- |
| Statistical test | Parameters | DF | F value | P value | Post-hoc Tukey HSD |
| 1-way-ANOVA | Pulsation | 2 | 905.2 | < 2 x 10^-16^ *** | No feeding – DOC (p = 0)  Phytoplankton – DOC (p = 0)  Phytoplankton – No feeding (p = 0) |
| 1-way-ANOVA | Growth  Rate | 2 | 2.848 | 0.0723 | n.a |
| 1-way-ANOVA | Symbio-diniaceae  density | 2 | 15.87 | 1.48 x 10^-5^ *** | No feeding – DOC (p = 0.0004)  Phytoplankton - DOC (p = 0.5)  Phytoplankton - No feeding (p = 0.00002) |
| 1-way-ANOVA | Mitotic  index | 2 | 5.144 | 0.0114 * | No feeding – DOC (p = 0.5)  Phytoplankton – DOC (p = 0.1)  Phytoplankton – No feeding (p = 0.009) |
| 1-way-ANOVA | Chloro-  phyll-*a*  per cell | 2 | 16.65 | 1x 10^-5^ *** | No feeding – DOC (p = 0.0000058)  Phytoplankton – DOC (p = 0.04)  Phytoplankton – No feeding (p = 0.008) |
| 1-way-ANOVA | Isotope  δ N15 | 2 | 0.571 | 0.571 | n.a |
| 1-way-ANOVA | Isotope  δ C13 | 2 | 0.698 | 0.505 | n.a |
| 1-way-ANOVA | Element  N % | 2 | 12.72 | 8.6 x 10^-5^ *** | No feeding – DOC (p = 0.00005)  Phytoplankton – DOC (p = 0.02)  Phytoplankton – No feeding (p = 0.07) |
| 1-way-ANOVA | Element  C % | 2 | 0.868 | 0.43 | n.a |
| 1-way-ANOVA | C : N | 2 | 21.7 | 1.48 x 10^-6^ *** | No feeding – DOC (p = 0.0000009)  Phytoplankton – DOC (p = 0.002)  Phytoplankton – No feeding (p = 0.01) |


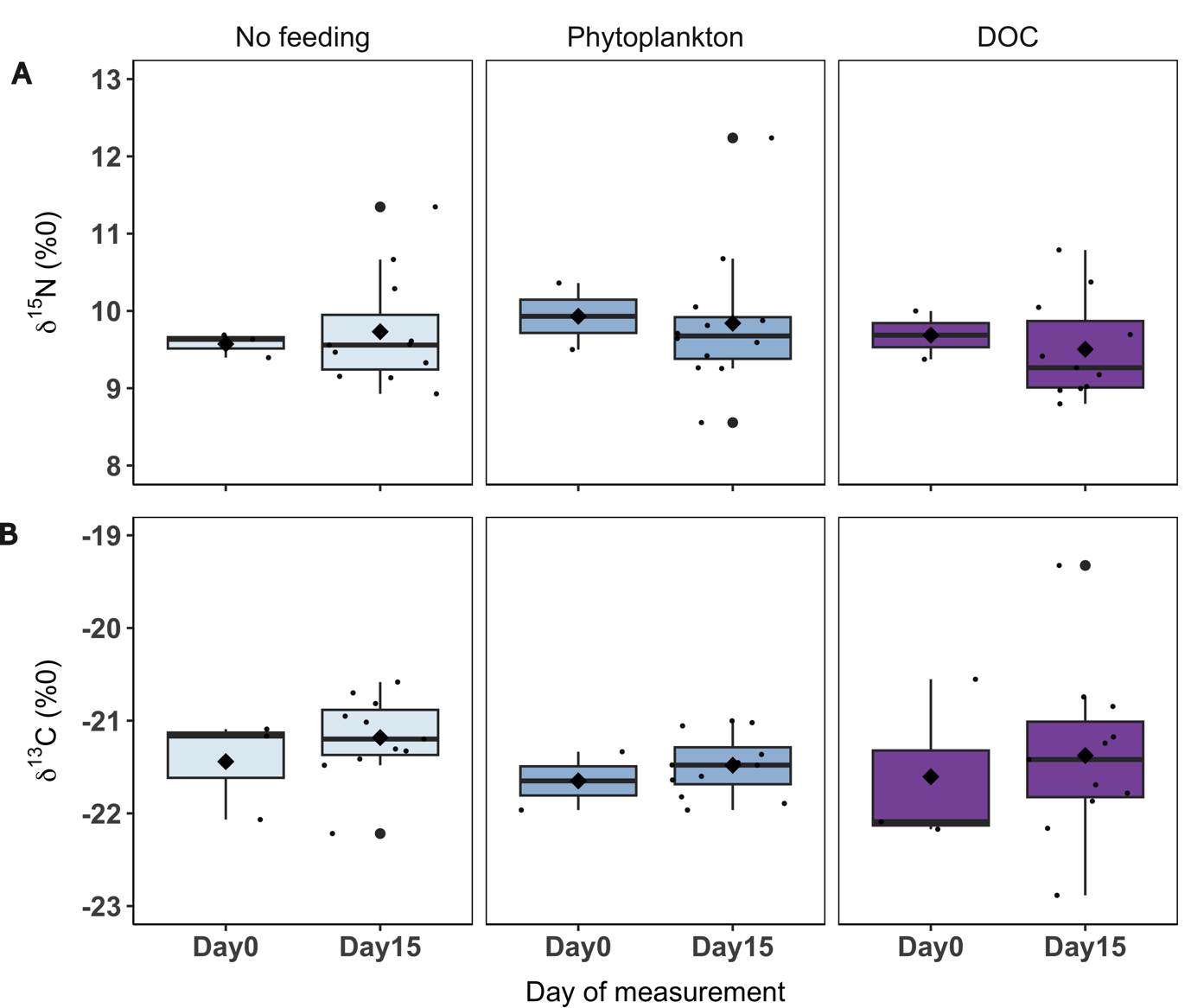


Figure S5 Non-significant stable isotope signatures of Xenia umbellata including A] nitrogen stable isotope ratio (δ^15^N (‰)) and B] carbon stable isotope ratio (δ^13^C (‰)), following 15 days of exposure to ‘No feeding’ ‘Phytoplankton’ and ‘Dissolved organic carbon [DOC]’ feeding treatments. For day 0 measurements, there are 3 biological replicates. For day 15 measurements there are 12 biological replicates. The median is represented by the black horizontal line and the mean is indicated by a large black diamond.
